# Diversity and Content of Carotenoids and Other Pigments in the Transition from the Green to the Red Stage of *Haematococcus pluvialis* Microalgae Identified by HPLC-DAD and LC-QTOF-MS

**DOI:** 10.3390/plants11081026

**Published:** 2022-04-09

**Authors:** Veno Jaša Grujić, Biljana Todorović, Roman Kranvogl, Terezija Ciringer, Jana Ambrožič-Dolinšek

**Affiliations:** 1 Department of Biology, Faculty of Natural Sciences and Mathematics, University of Maribor, Koroška 160, 2000 Maribor, Slovenia; veno.grujic@um.si (V.J.G.); terezija.ciringer@um.si (T.C.); 2 Department of Elementary Education, Faculty of Education, University of Maribor, Koroška 160, 2000 Maribor, Slovenia; 3 Department of Botany and Plant Physiology, Faculty of Agriculture and Life Sciences, University of Maribor, Pivola 10, 2311 Hoče, Slovenia; biljana.todorovic@um.si; 4 Centre for Chemical Analysis of Food, Water and Other Environmental Samples, National Laboratory of Health, Environment and Food, Prvomajska 1, 2000 Maribor, Slovenia; roman.kranvogl@nlzoh.si

**Keywords:** antioxidants, astaxanthin, chlorophylls, bioactive compounds, astaxanthin, algae, *Haematococcus*, life cycle, pigment composition, secondary carotenoids

## Abstract

*H. pluvialis* is a unicellular freshwater alga containing many bioactive compounds, especially carotenoids, which are the strongest antioxidants among the pigments. This study evaluates the composition and content of carotenoids and other pigments in both stages of algae life cycle, especially in the green vegetative stage, less studied in comparison to the red stage. To determine the composition and content of carotenoids, a combination of HPLC-DAD and LC-QTOF-MS was used. The content of carotenoids in the green vegetative stage was significantly lower than in the red vegetative stage. In the green vegetative stage, 16 different carotenoids and other pigments were identified. Among the total 8.86 mg g^−1^ DW of pigments, 5.24 mg g^−1^ DW or 59% of them were chlorophyll *a* with its derivatives, and 3.62 mg g^−1^ DW or 41% of them were free carotenoids. After the transition from the green to the red stage, the carotenoid composition was replaced by secondary carotenoids, astaxanthin and its esters, which predominated in the whole carotenoid composition. In addition to free astaxanthin, 12 astaxanthin monoesters, 6 diesters and 13 other carotenoids were determined. The majority of 37.86 mg g^−1^ DW pigments were monoesters. They represented 82% of all pigments, and their content was about 5 times higher than both, diesters (5.91 mg g^−1^ DW or 12% of all) and free carotenoids (2.4 mg g^−1^ DW or 6% of all). The results of the study contribute to the data on the overall pigment composition and content of *H. pluvialis* algae and provide the basis for further improvement of cultivation of the *H. pluvialis* algae.

## 1. Introduction

Antioxidants are bioactive compounds that can prevent, or slow cell damage caused by free radicals [1], which is why they are of great importance for human and animal health [2]. One of the most important groups of compounds with strong antioxidant properties are carotenoids, which are mainly used in food, cosmetic and pharmaceutical industries [3]. Carotenoids are fat-soluble pigments that are generally classified into two main groups, oxygen-containing xanthophylls (e.g., astaxanthin, zeaxanthin) and oxygen-free carotenes (e.g., β-carotene and lycopene) [4]. Chlorophylls are another group of more than 100 different structures that are primarily associated with photosynthesis. There is also some evidence, that chlorophylls have antioxidant properties [5] and affects secondary metabolism including carotenoid synthesis [6].The antioxidant properties of both groups of pigments, chlorophylls and carotenoids, have been investigated and recently reviewed by several authors [5,7,8].

Microalgae synthesize a variety of bioactive compounds, including carotenoids and all known xanthophylls found in plants [9]. They can synthesize a variety pigments (e.g., astaxanthin) that are specific only to algae, cyanobacteria, and some other organisms [10]. According to Novoveská et al. [4], carotenoids can be divided into two groups: primary carotenoids, which are components of the photosynthetic apparatus and are essential for survival, and secondary carotenoids, which are produced via carotenogenesis only when cells are exposed to specific environmental conditions, such as high light radiation, nutrient deficiency, salinity and other stress factors [11]. 

*H. pluvialis* (*Volvocales*, *Chlorophyceae*, *Chlorophyta*) is a freshwater green microalga containing many bioactive compounds such as carotenoids, proteins, lipids, carbohydrates and other substances [12,13,14]. It is considered one of the richest sources of xanthophyll carotenoids, astaxanthin [15,16,17], which is a very valuable and important industrial pigment [18]. In addition to astaxanthin, *H. pluvialis* also contains other carotenoids, such as cantaxanthin, lutein, β-carotene, α-carotene, β-cryptoxanthin, lycopene, lutein, violaxanthin and others [13,19,20]. 

The composition and content of carotenoids in microalga *H. pluvialis* vary according to the development of its life cycle, which is divided into two stages: the first refers to a green motile vegetative stage (macrozooids, microzooids and palmela), in which the microalgal cells continuously divide, grow and synthesize chlorophylls and carotenoids. The second refers to a red nonmotile stage (macrozooid), where cell division stops and the content of secondary metabolites, especially astaxanthin and its esters, increases [15,21]. The red stage is stimulated by stressors such as nutrient deficiency, high temperature and high light intensity, among others [14].

Carotenoids have been quantified by various analytical methods, such as HPLC-DAD [11,22,23], LC-(APCI) MS [24,25] and LC-QTOF-MS [26,27]. Some authors reported that more effective extraction is possible by using nonpolar solvents, depending on the target group of carotenoids [28,29]. However, the analysis of carotenoid esters is complex and challenging due to their polarity, instability and diversity [26,30,31]. In addition, some interfering compounds can cause high background noise and ionization suppression in mass spectrometric analysis. Metlicar et al. [32] state that the choice of an appropriate solvent in the initial isolation of xanthophylls is crucial for the efficiency of esterification. They note that the use of nonpolar solvents such as β-pinene and sc-CO_2_ causes less reaction interference. Todorović et al. [26] compared the extraction efficiency of different solvents, where MTBE was found to be the most suitable solvent for the extraction and identification of carotenoids in the red stage, especially esters. In contrast to the red stage, the extracts obtained with ethanol showed the highest yield and antioxidant activity in the green stage [33].

Published research has mainly focused on the red development stage and the identification of astaxanthin and its esters [3,13,21] and less on the green vegetative stage [6,14]. Approximately 70–79% monoesters, 20–25% diesters, and only 1–5% of the free astaxanthin is present in the red cells of alga *H. pluvialis* [14,26]. Alga *H. pluvialis* in the green vegetative stage consists mainly of lutein, β-carotene [14], primary carotenoids (violaxanthin, neoxanthin) [34,35], and other pigments such as chlorophyll *a* and *b* [14,25,33,34,35,36]. 

Several data about the composition and content of carotenoids and other pigments, not only in the green but also in red developmental stages, have been obtained by low resolution methods [33,34,35,36]. Due to the great interest in determining the carotenoid and other pigment compositions and content of the microalga *H. pluvialis*, the aim of our study was to improve our current understanding of the biochemistry of the transition from the green to the red stage during its life cycle. Therefore, the objective of the study was to separate, identify, compare, and evaluate the carotenoids and other pigments in both developmental stages of the life cycle of the alga *H. pluvialis,* with a combination of HPLC-DAD and LC-QTOF-MS methods. 

The study focused not only on the astaxanthin and its esters but also on a whole range of pigments of the green vegetative stage, in order to improve the data on the overall pigment composition and content of *H. pluvialis*.

## 2. Results

### 2.1. Carotenoids and Other Pigments in Green Vegetative Stage of Microalga H. pluvialis

**Figure 1:**
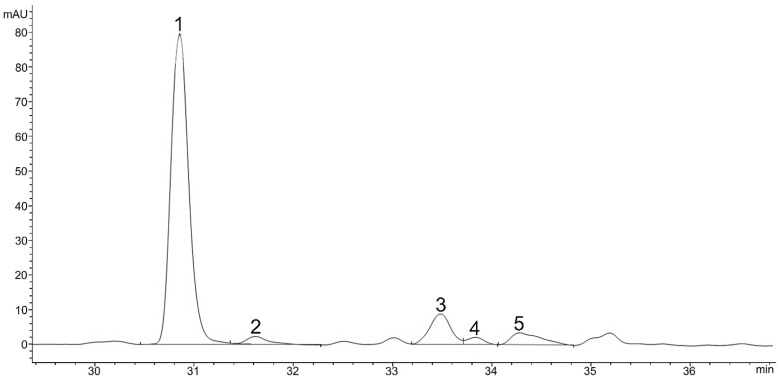
HPLC chromatogram (DAD, 475 nm) of chlorophylls in green vegetative stage, Chlorophyll *b* (1), Chlorophyll *b* derivate (2), Chlorophyll *a* (3), Chlorophyll *a* derivate (4), Echinenone (5).

**Figure 2:**
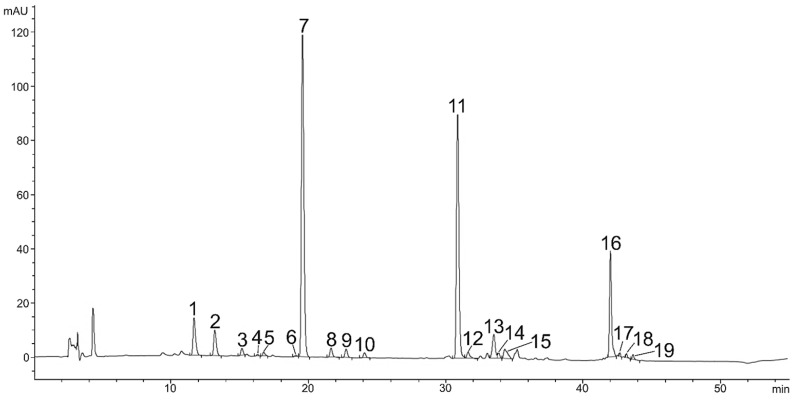
HPLC chromatogram (DAD, 475 nm) of chlorophylls and carotenoids in green vegetative stage, Neoxanthin (1), Violaxanthin (2), Astaxanthin (3), Adonixanthin (4), Astaxanthin isomer (5), Zeaxanthin (6), Lutein (7), Lutein isomer (8), Lutein isomer (9), Lutein isomer (10), Chlorophyll *b* (11), Chlorophyll *b* derivate (12), Chlorophyll *a* (13), Chlorophyl *a* derivate (14), Echinenone (15), *B-*Carotene (16), Not identified (17), Not identified (18), Not identified (19).

A total of 16 carotenoids and other pigments were separated from the green vegetative stage (Table 1). Carotenoids and other pigments were listed and identified in order of retention time during elution through a C18 column as neoxanthin, violaxanthin, astaxanthin, adonixanthin, antheraxanthin, zeaxanthin, lutein isomer 1, lutein isomer 2, lutein isomer 3, chlorophyll *a*, chlorophyll *b*, chlorophyll *a* derivate, chlorophyll *b* derivate, echinenone, and β-carotene. During the 44 min separation, all xanthophylls appeared in the first 11.6–24.1 min (Figure 1), chlorophylls in the next 30.9–35.2 min and other carotenoids after 42 min (Figure 2). The retention time of xanthophylls (11.6–24.1) was shorter than the retention time of the chlorophyll b (30.9), chlorophyll *b* derivate (31.6), chlorophyll *a* (30.5), and chlorophyll *a* derivate (33.7). β-carotene (42) had the longest retention time (Figure 2). 

Using HPLC-DAD, we determined the highest content of chlorophyll *a* (4.58 ± 0.56 mg g^−^^1^) with its derivate (0.66 ± 0.17 mg g^−^^1^). Xanthophylls lutein (1.12 ± 0.09 mg g^−^^1^) and β-carotene (0.89 ± 0.04 g^−^^1^) present most of the carotenoid fraction, mainly 32% of lutein and 25% of β-carotene. The content of xanthophylls adonixanthin (0.17 ± 0.00 mg g^−^^1^), antheraxanthin (0.04 ± 0.00 mg g^−^^1^) and neoxanthin (0.44 ± 0.04 mg g^−^^1^) was lower. The content of free astaxanthin (0.06 ± 0.01 mg g^−^^1^) and echinenone (0.06 ± 0.01 mg g^−^^1^) was nearly the same and presents the minority of the pigment fraction. The total content of pigments was 8.86 mg g^−^^1^, containing 5.24 mg (59%) of the chlorophyll *a* with its derivatives and 3.62 mg g^−^^1^ (41%) of carotenoids. Chlorophyll *b* and its derivatives were identified using HPLC-DAD and LC-QTOF-MS, but due to the lack of a chlorophyll *b* reference standard, it was not possible to perform quantification.

### 2.2. Carotenoids in Red Stage of Microalga H. pluvialis

Free astaxanthin was detected as a quasimolecular ion at *m/z* 597.3938 [M+H]^+^. Esterified forms, astaxanthin fatty acid monoesters were assigned as [M+H]^+^ and their fragmentation products as [M+H-FA]^+^. Other esterified forms, astaxanthin diesters, were also determined as *m/z* [M+H]^+^ and their fragmentation products. The location of the double bonds was not identified because mass diversions between quasimolecular and fragment ions were used to assign the acyl chains. 

**Figure 3:**
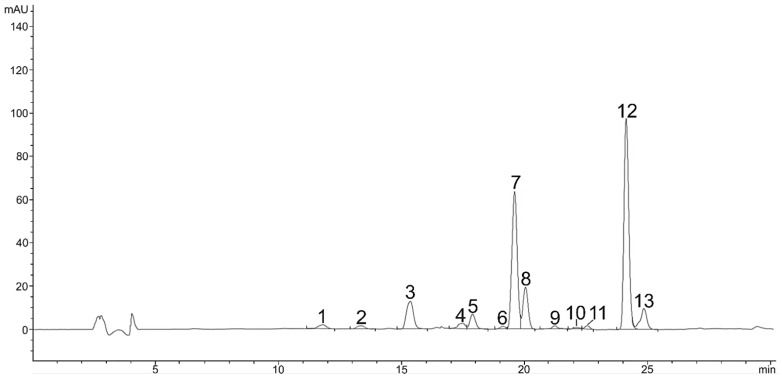
HPLC chromatogram (DAD, 475 nm) of free carotenoids in red stage, Neoxanthin (1), Violaxanthin (2), Astaxanthin (3), Adonixanthin (4), Not identified (5), Zeaxanthin (6), Lutein (7), Adonirubin (8), Lutein isomer (9), Lutein Isomer (10), Lutein isomer (11), Cantaxanthin (12), Cis-cantaxanthin (13).

**Figure 4:**
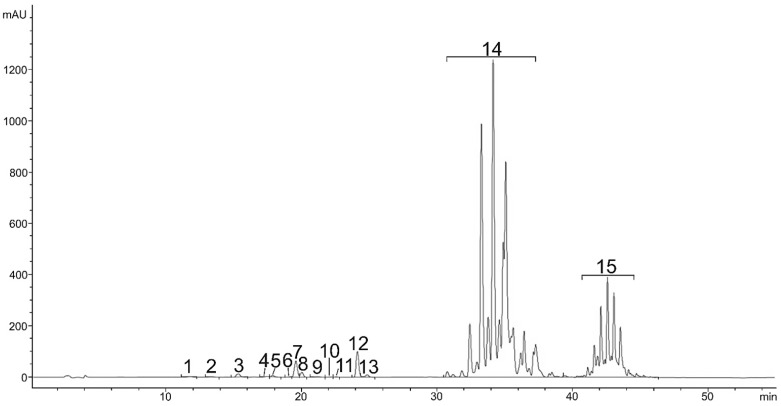
HPLC chromatogram (DAD, 475 nm) of all carotenoids in red stage, Neoxnathin (1), Violaxanthin (2), Astaxannthin (3), Adonixanthin (4), Not identified (5), Zeaxanthin (6), Lutein (7), Adonirubin (8), Lutein isomer (9), Letein isomer (10), Lutein isomer (11), Cantaxanthin (12), Cis-Cantaxanthin (13), Monoesters (14), Diesters (15).

**Figure 5:**
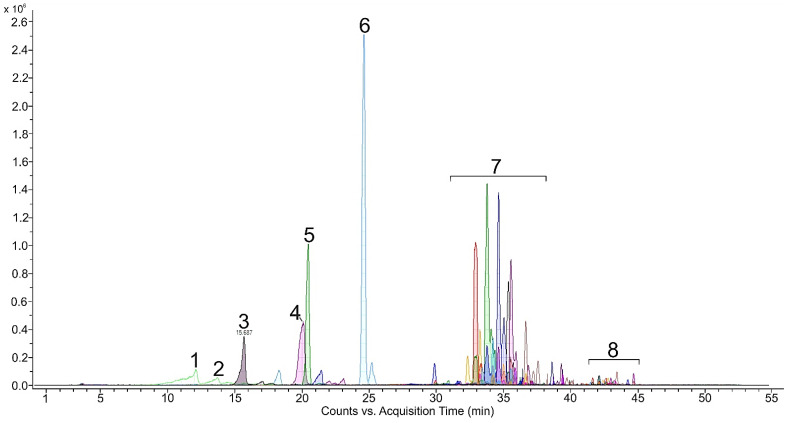
LC-QTOF-MS chromatogram from *H. pluvialis* sample, extracted masses of Neoxanthin (1), Violaxanthin (2), Astaxanthin (3), Lutein (4), Adonirubin (5), Canthaxanthin (6), Monoesters (7) and Diesters (8).

**Figure 6:**
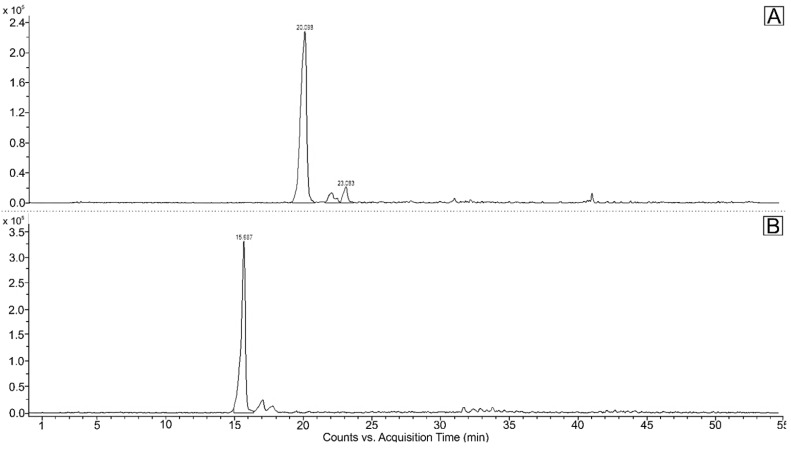
LC-QTOF-MS extracted chromatogram of red stage at 569.4353 [M+H]^+^ for Lutein and Lutein isomers (**A**), and 597.3938 [M+H]^+^ for Astaxanthin (**B**).

Carotenoid composition of the green vegetative stage was replaced by secondary carotenoids. They consisted of astaxanthin and its esters, which predominated in the whole carotenoid composition. The content of astaxanthin and its esters in the red stage was significantly higher. Most detected astaxanthin was presented in bound monoester or diester forms. Beside free astaxanthin, 12 astaxanthin monoesters, 6 diesters and 11 other carotenoids were determined (Table 2). Carotenoids and other pigments were listed and identified in order of retention time during elution through a C18 column as neoxanthin, violaxanthin, astaxanthin, adonixanthin, antheraxanthin, zeaxanthin, lutein, adonirubin, lutein isomer 1, lutein isomer 2, lutein isomer 3, canthaxanthin, cis-canthaxanthin, and echinenone. During the 46 min separation, all xanthophylls appeared in the first 11.6–24.8 min (Figure 3), astaxanthin monoesters in the next 31.6–38.0 min and astaxanthin diesters after 40.6 min (Figure 4). These results are also consistent with the LC-QTOF-MS-extracted chromatogram (Figure 5) of the red stage. Figure 6 shows LC-QTOF-MS-extracted chromatogram of the red stage at 569.4353 [M+H]^+^ for lutein and lutein isomers (A) and 597.3938 (M+H)**^+^** for astaxanthin (B). The ratio of relative retention times between lutein and lutein isomers is consistent with the results of other authors [37]. 

The content of astaxanthin monoesters ranged from 0.11 ± 0.00 to 8.61 ± 0.38 mg g^−^^1^ DW. The highest content had M+H-C16:0 (8.61 ± 0.38 mg g^−^^1^), which is 78-times higher than the content in M+H-C18:1 (0.11 ± 0.00 mg g^−^^1^). Concentration of astaxanthin diesters ranged from 0.19 ± 0.00 to 2.24 ± 0.09 mg g^−^^1^ DW. The highest content had M+H-C18:1/C18:3 (2.24 ± 0.09 mg g^−^^1^), which is 12-times higher than content in M+H-C18:2/C18:3 (0.19 ± 0.00 mg g^−^^1^). Free astaxanthin reached 0.23 ± 0.04 mg g^−^^1^. The total content of carotenoids was 46.2 mg g^−^^1^, of which free carotenoids reached 2.43 mg g^−^^1^ (5%), astaxanthin monoesters 37.86 mg g^−^^1^ (82%) and astaxanthin diesters 5.91 mg g^−^^1^ (13%).

## 3. Discussion

The study was performed with a strain that has not been analyzed before and is available to the entire scientific and public community. We have analyzed and published the whole life cycle of *H. pluvialis* in a single study of the green and red stages of the same strain, which has also never been conducted before. The results, obtained with the current methods complement those obtained with several other older and sometimes low-resolution methods [33,34,35,36]. In addition, they provide new insights into the composition and content of carotenoids and other pigments with emphasis on the less studied green stage of the microalga *H. pluvialis*.

### 3.1. Green Vegetative Stage

Data on the composition of pigments in the green phase are important because they can indirectly influence the synthesis of secondary metabolites in the red phase. The green vegetative stage consists of light harvesting pigments, divided into primary light harvesting (chlorophyll *a*) and accessory light harvesting pigments (chlorophylls and some carotenoids) [8]. Chlorophyll *a,* as a primary light harvesting pigment, transfers excitation energy directly to the photosynthetic electron transport chain, while other chlorophyll *a* and b transfer their excitation energy via chlorophyll *a* [38]. Therefore, a green stage is indicated with the highest chlorophyll *a* content. The concentration of chlorophyll *a* and its derivatives in our extract reached 5.24 mg g^−1^ or 59% of all detected pigments, in comparison, the study of Grewe and Griehl [39] determined only 2% chlorophyll of total pigments. Dragoş et al. [40] also analyzed a high total chlorophyll content (17.8 mg g^−1^), which accounted for 84% of all identified pigments in the green stage. Compared to other studies, a higher proportion of chlorophyll *a* and approximately the same proportion of lutein and β-carotene were determined [3,14,33,36,41]. 

Other pigments of the green stage, such as carotenoids, transfer excitation energy via chlorophyll *a* [38] and protect photosystems against excess light via several mechanisms of nonphotochemical quenching (NPQ) [42]. The highest proportion of lutein (32%) and β-carotene (23%) in the carotenoid fraction corresponded to the proportions of lutein (40–56%) and β-carotene (17–30%) of other studies [3,14,33,36,41]. Although lutein is known as a photoprotection carotenoid, it is also involved in the light harvesting process [8]. Furthermore, it can not only transfer excitation energy to chlorophyll *a* with very low efficiency [43], but also has a photoprotection role. Some carotenoids, such as astaxanthin [40], antheraxanthin, violaxanthin, zeaxanthin [44], zeaxanthin and neoxanthin [14,39], were identified in the green vegetative stage. 

Carotenoids present in lower concentrations are involved in photoprotection (NPQ) [42], filtering, quenching, or scavenging the light energy [8]. Astaxanthin and β-carotene prevent the overexcitation of chlorophyll *a* by absorbing excess radiation [8]. Astaxanthin, β-carotene, lutein, zeaxanthin, and violaxanthin are quenchers [8]. The role of astaxanthin, β-carotene, lutein, zeaxanthin, and neoxanthin [45] is related to the prevention of cell damage by scavenging free radicals and reacting with reactive oxygen species (ROS). In addition to light harvesting, violaxanthin, zeaxanthin, and lutein also have a photoprotective function. The presence of some carotenoids in the green stage, such as astaxanthin and echinenone, most likely indicates the end of the green vegetative stage and the transition to the red stage. 

Although the red stage of *H. pluvialis* is economically more interesting because of the accumulation of astaxanthin, the control of the initial green vegetative stage also plays an important role in its further biosynthesis. Cell properties in the green proliferation stage play an important role in photoinduction in the red stage [6]. With sequential heterotrophy dilution photoinduction (SHDP) technology, it was found that the high accumulation capacity of astaxanthin was associated with high chlorophyll content in cells *H. pluvialis*. With additional transcriptome analysis, Fang et al. [6] found that these were associated with increased astaxanthin synthesis regulation genes and decreased chlorophyll and lutein synthesis regulation genes. Moreover, the chlorophyll degradation product could also be used for the synthesis of astaxanthin. Jayara et al. [46] investigated the metabolic engineering of novel ketocarotenoid production in carrot plants. The synthesis of ketocarotenoids was achieved by the introduction and upregulation of enzymes catalyzing the conversion steps from β-carotene to astaxanthin, and 70% of β-carotene was converted to ketocarotenoids [46]. However, the extract from the green stage had the highest content of chlorophyll, lutein, and β-carotene. Further synthesis of secondary carotenoids, especially astaxanthin, depends mainly on the content of β-carotene, which is the main precursor of astaxanthin and other secondary carotenoids.

### 3.2. Red Stage

After unfavorable conditions, the life cycle of *H. pluvialis* completely changed. Metabolism was redirected to the accumulation of secondary pigments. The primary carotenoids of the green stage were replaced by other secondary carotenoids, which has also been reported by other authors [35,40]. The secondary pigments were then esterified; free forms were converted into esterified forms. Astaxanthin was the only free carotenoid whose content increased compared to the green phase, which is consistent with the results of other studies [3,14,33,35,41]. The content of chlorophylls decreased and vanished from the red stage. 

Esterification in plants is thought to increase the chemical stability of the core of carotenoids and facilitate their migration from disrupted chloroplasts into chromoplasts [32,47]. The process increases the lipophilicity of xanthophylls, which affects the formation of specialized structures in chromoplasts and increases photoprotection. Esterification is also associated with color changes during fruit ripening [47]. Similarly, esterification in *H. pluvialis* also decreases chloroplast volume and increases the content of esters throughout the cell. Most of the astaxanthin and its esters are deposited as mono- or diesters in the cytosolic lipid bodies during the red stage of the *H. pluvialis* life cycle. According to Shaah et al. [14], this type of modification allows the deposition of this polar molecule in the nonpolar matrix of lipid droplets. However, the biosynthesis of astaxanthin and its esters is upregulated under stress conditions [14].

Among the 31 carotenoids identified in our study, 12 were classified as monoesters, accounting for 82% of all pigments, and six were diesters, accounting for 12% of pigments. Only 13 were free carotenoids, representing 6% of all pigments. The content of chlorophylls and carotenoids (neoxanthin, violaxanthin, zeaxanthin, adonixanthin, and lutein) decreased significantly compared to the green stage. The content of free carotenoids in the red stage was only 2.4 mg g^−1^ DW and dropped by 35% to a final 6% of all pigments. Astaxanthin in free form represented only 0.23% of all pigments. This is in agreement with the results of Todorović et al. [26], who identified 11 astaxanthin monoesters, 79% of all pigments, six astaxanthin diesters (20%) and a small 1% of other carotenoids. Sarada et al. [48] reported about 0.5% free astaxanthin, 72.0% monoester and 27.5% diesters.

Using HPLC-(+)APCI-MS/MS, Zhou et al. [28], in their samples, observed slightly higher diversity of astaxanthin molecules: 20 astaxanthin esters, eight monoesters and 12 diesters. Using LC-(APCI)MS, Miao et al. [24] determined four free carotenoids, 15 astaxanthin monoesters, and 12 astaxanthin diesters. Their identification of the compounds was based on the characteristic fragment ions of the negative ion mode, positive ion mode and MS2. Astaxanthin monoesters in the positive ion mode showed that *m/z* 597.4 and 579.4 of peak 1 protonated quasimolecular ions [M+H]^+^ and [M+H-18]^+^ of the mass spectra, which is in line with our results. In addition, an example of a mass spectrum obtained from an astaxanthin monoester (M+H-C18:3) in *H. pluvialis* extract exhibits the same spectral characteristics as in the case of our study. The *m/z* 857.7 was the fragment of protonated quasi-molecular ions. The situation was similar for astaxanthin diesters (M+H-C18:3/C18:2), where *m/z* 1119.8 represented the fragment of protonated quasi-molecular ions.

## 4. Materials and Methods

### 4.1. Microalgal Culture and Growth Conditions

Axenic *H. pluvialis* (*Volvocales*, *Chlorophyceae*, *Chlorophyta*) strain 1081 was provided by the Culture Collection of Autotrophic Organisms (CCALA). Culture suspensions were initiated from agar–agar stock cultures in 250 mL of Bold’s Basal Medium (BBM) [49,50]. After 14 days, the algal suspension was reinoculated into 15 Erlenmeyer flasks containing 250 mL BBM at an initial algal density of 20 mg L^−1^. Culture conditions were 25 ± 1 °C, 16 h/8 h light/dark cycle [21] at a light intensity of 140 µmol photons m^−2^ s^−1^ in the green stage during the first 15 days, and of 280 µmol photons m^−2^ s^−1^ in the red stage during the second 15 days. All Erlenmayer flasks were illuminated from below. Cultures were aerated with sterile humidified air (1.85 L air per L^−1^ culture medium min^−1^) with the addition of 1.5% carbon dioxide (CO_2_) and shaken three times daily. To check the purity of the algal cultures, a solid medium was prepared with an additional 1% peptose, glucose and yeast extract. Green motile cells were analyzed after 15 days. Red algal cells were analyzed after 30 days of cultivation.

### 4.2. Dry Weight Determination

We determined the dry weight (DW) of the microalgal biomass based on the differences in the dry weight of the filters before and after filtration of the algal suspension [51]. We dried Whatman GF/C glass fiber filters at 105 °C for 2 h, cooled them in a desiccator, and weighed them. After filtration, we repeated the whole process: drying, cooling and weighing. The density of the suspension was determined as mg L^−1^ DW.

### 4.3. Carotenoid Extraction

Samples of 5 mL algal suspension were centrifuged at 4000 rpm for 5 min. The Ticks and rigid cell walls of the red algal cells were disrupted with Hydrochloric acid (HCl) [48]. The pellets were treated with 2 mL of 1 M HCl at 70 °C for 2 min in a heating block, cooled, and centrifuged at 4000 rpm for 5 min. The sediments were washed twice with 2 mL of deionized water and prepared for carotenoids extraction using tert-butyl methyl ether (MTBE) from Fluka Chemie (Buchs, Switzerland). To prevent oxidation of the carotenoids, 1% of butylated hydroxytoluene (BHT) was added.

The carotenoids were extracted by 5 mL of MTBE [26]. Glass beads with a diameter of 0.4 mm were added to 5 mL of the solvent, vortexed for 5 min and centrifuged at 3000 rpm for 3 min. The same procedure was performed for the green vegetative stage, but without HCL pre-treatment and using ethanol (ETA) as the solvent instead of MTBE. The extraction was repeated at room temperature until the sediment was completely colorless, but no longer than 20 min. The efficiency of the extraction was verified by examining the color of the broken cells under a light microscope. The extracts were stored in a dark place at −20 °C.

### 4.4. HPLC-DAD and LC-QTOF-MS Analysis of Carotenoids

Analyses were performed at the National Laboratory of Health, Environment and Food (NLZOH), Maribor. Pigments were separated using an Agilent 1260 HPLC system (Agilent technology, Santa Clara, CA, USA) coupled with a DAD detector. The spectrum was recorded from 200 to 800 nm. The wavelength for quantitative parameters was 200–500 nm. The column was Infinity Lab Poroshell 120 EC-C18: 150 × 3.0 mm, 2.7 µm, PN: 693975-302(T), SN: USCFW11265; the column temperature was 30 °C, the injection volume was 10.00 µL, the analysis time was 55 min. The method was modified according to Hrvolová et al. [52]. Mobile stages (A): 0.1% (*v*/*v*) formic acid in 50% ultrapure water and 50% methanol; (B): 0.1% (*v*/*v*) formic acid in 80% methyl tert-butyl ether and 20% methanol. The eluent flow was 0.2 mL min^−1^. The gradient started at 35% eluent B, changed to 90% eluent B over 40 min, held for 4 min and returned back to 35% eluent B. Equilibration lasted for 10 min. Spectral data were processed using Open Lab software. Substance identification was performed in several steps and was confirmed by comparing retention times with standard solution and spectral properties of the substance. The content of carotenoids substances was expressed in mg g^−1^ DW. The individual bioactive compound identification was performed by using an Agilent 6530 LC-QTOF mass spectrometer with electrospray ionization (ESI) set to positive ionization. The compounds were analyzed over the entire mass range (from 50–1700 *m/z*). The chromatographic conditions and column settings were the same as for the HPLC-DAD analysis. The sample injection volume was 10.00 µL and the flow rate was 0.2 mL min^−1^. MS parameters [26] were 4 GHz, high resolution was a maximum of 1700 *m/z*, acquisition rate 1.0 spectra/s. Sample ionization was Dual ESI, and ion source positive ion scan mode used mass scanning from 50 to 1700 *m/z*. Other LC- QTOF parameters: drying gas (N2) and temperature 300 °C; drying gas flow rate 10 L/min; nebulizer 40 psig; Vcap. 4000 V; nozzle 2000 V; skimmer 65 V; fragmentor 175 V and Octopole RF 750 V. Spectral data were processed by using quality Agilent Mass Hunter Qualitative Software and Agilent PCDL Manager. Substance identification was performed by a comparison of retention times with standard solutions, the determination of accurate mass and the fragmentation of compounds. The contents of bioactive substances were expressed in mg g^−1^ DW. HPLC-grade methanol (J. T. Baker, Phillipsburg, NJ, USA) and MTBE were purchased from Sigma Aldrich, USA. The formic acid (≥99%) for LC-MS was purchased from VWR Chemicals (Chicago, IL, USA). 

### 4.5. HPLC Standards

Standards Chlorophyll *a*, analytical standard, PN: 96145, BN: BCCF9581, Violaxanthin, analytical standard, PN: 91904, BN: BCCG6377, Lutein, analytical standard, PN: 07168, BN: BCCG1715, 9-cis-Anteraxanthin, analytical standard, PN: 47999, BN: BCCG6378, Echinenone, analytical standard, PN: 73341, BN: BCCF6266, Zeaxanthin, analytical standard, PN: 14681, BN: BCCD7729, Neoxanthin, analytical standard, PN: 72994, BN: BCCC9416, β-Carotene, analytical standard, PN: 22040, BN: 100919781 were purchased from Sigma Aldrich (Saint Louis, MO, USA).

### 4.6. Method Validation

The analytical method was validated in terms of repeatability, linearity, accuracy and stability. Linearity was checked at five concentration levels. An external method was used for quantification. Linear regression analysis was used to calculate the slope, intercept and the correlation coefficient of each calibration line. LOD and LOQ were calculated from S/N, for LOQ >10. Selectivity was demonstrated by the analysis of blank and was assessed by the absence of interference in the same chromatographic analysis. Repeatability was estimated by six replicate measurements at the same concentration levels. The precision (RSD) was studied by analyzing spiked samples at three concentration levels by expressing the SD of repeated measurements as the percentage of the mean value. For the stability study, three samples were analyzed immediately, and the rest were stored at −20 °C. Stability was assessed within a period of 3 months of storage at −20 °C. The 10% degradation criterion was used to evaluate stability.

### 4.7. Statistical Analysis

Results were statistically analyzed using SPSS^®^ 27 software (SPSS Inc., Chicago, IL, USA) by one-way analysis of variance (ANOVA). The level of statistical significance (*p*) of differences between the content of green (*n* = 15) and red (*n* = 15) stages of the algal life cycle was determined using Duncan’s post hoc test. Differences at *p* ≤ 0.05 were considered statistically significant. Each treatment was repeated five times.

## 5. Conclusions

Our study provides new insights into the composition and content of carotenoids and other pigments, not only in the red but also in the green developmental stage, using current methods. The study was conducted with a strain that has not been analyzed before and is available to the entire scientific and public community. Unlike some other studies, both the green and red stages were analyzed using the same strain.

The composition and content of carotenoids and other pigments were determined before and after transition from the green vegetative to the red stage of the culture model of *H. pluvialis*. Data on the composition of pigments in the green stage are important because they can indirectly influence the synthesis of secondary metabolites in the red phase. Therefore, the focus was on the transition from primary carotenoids to secondary carotenoids and their transition to esterified forms. Free astaxanthin was the only free carotenoid whose content increased. Reliable methods and analyses performed at HPLC-DAD and LC-MS-QTOF allowed us to properly separate and identify astaxanthin, lutein, β-carotene, and other bioactive compounds at both stages of the developmental cycle, complementing previous studies.

## Figures and Tables

**Table 1:** Identification and content of pigments, and their spectral characteristics in the green vegetative stage of the alga *Haematococcs pluvialis* determined by HPLC (mean ± SE).

No.	Compound Name	Content (mg g^−1^ DW)	Rt (min) HPLC-DAD	*m/z*	Adduct
1	Neoxanthin	0.44 ± 0.04	11.6	601.4251	[M+H]^+^
2	Violaxanthin	0.57 ± 0.07	13.1	601.4251	[M+H]^+^
3	Astaxanthin	0.06 ± 0.01	15.1	597.3938	[M+H]^+^
4	Adonixanthin	0.17 ± 0.00	16.3	583.4362	[M+H]^+^
5	Antheraxanthin	0.04 ± 0.00	16.7	585.4302	[M+H]^+^
6	Zeaxanthin	0.01 ± 0.00	19.1	569.4353	[M+H]^+^
7	Lutein	1.12 ± 0.09	19.5	569.4353	[M+H]^+^
8	Lutein isomer 1	0.02 ± 0.01	21.6	569.4353	[M+H]^+^
9	Lutein isomer 2	0.04 ± 0.00	22.7	569.4353	[M+H]^+^
10	Lutein isomer 3	0.02 ± 0.00	24.1	569.4353	[M+H]^+^
11	Chlorophyll *b*	/	30.9	9075.219	[M+H]^+^
12	Chlorophyll *b* derivate	/	31.6	9075.219	[M+H]^+^
13	Chlorophyll *a*	4.58 ± 0.56	33.5	8935.426	[M+H]^+^
14	Chlorophyll *a* derivate	0.66 ± 0.17	33.7	895.5219	[M+H]^+^
15	Echinenone	0.06 ± 0.01	34.2	551.4247	[M+H]^+^
16	β-Carotene	0.89 ± 0.04	42.0	537.4455	[M+H]^+^

*m/z*: ratio of an ion’s mass (m) in atomic mass units (z) to its formal charge (z); M: molecule with molecular weight m; Adduct: product of a direct.

**Table 2:** Identification and content of pigments, and their spectral characteristics in the red phase of the alga *Haematococcs pluvialis* determined by HPLC (mean ± SE).

No.	Compound Name	Content (mg g^−1^ DW)	Rt (min) HPLC-DAD	*m/z*	Adduct	Product Ion (M-FA)
1	Neoxanthin	0.12 ± 0.03	11.8	601.4251	[M+H]^+^	/
2	Violaxanthin	0.18 ± 0.05	13.3	601.4251	[M+H]^+^	/
3	Astaxanthin	0.23 ± 0.04	15.1	597.3938	[M+H]^+^	/
4	Adonixanthin	0.05 ± 0.01	16.6	583.4362	[M+H]^+^	/
5	Zeaxanthin	0.02 ± 0.00	19.1	569.4353	[M+H]^+^	/
6	Lutein	0.55 ± 0.14	19.5	569.4353	[M+H]^+^	/
7	Adonirubin	0.18 ± 0.03	20.0	581.3989	[M+H]^+^	/
8	Lutein isomer	0.18 ± 0.01	21.2	569.4353	[M+H]^+^	/
9	Lutein isomer	LOQ	21.0	569.4353	[M+H]^+^	/
10	Lutein isomer	LOQ	22.5	569.4353	[M+H]^+^	/
11	Canthaxanthin	0.80 ± 0.17	24.1	565.4040	[M+H]^+^	/
12	Cis-Canthaxanthin	0.12 ± 0.03	24.8	565.4040	[M+H]^+^	/
13	M+H-C16:2	1.69 ± 0.07	/	831.5928	[M+H]^+^	579.3840
14	M+H-C16:1	7.69 ± 0.29	/	833.6084	[M+H]^+^	579.3840
15	M+H-C16:0	8.61 ± 0.38	/	835.6241	[M+H]^+^	579.3840
16	M+H-C18:4	0.31 ± 0.01	/	855.5928	[M+H]^+^	579.3840
17	M+H-C18:3	1.69 ± 0.08	/	857.6084	[M+H]^+^	579.3840
18	M+H-C18:2	7.04 ± 0.06	/	859.6241	[M+H]^+^	579.3840
19	M+H-C20:2	1.57 ± 0.36		883.6241	[M+H]^+^	579.3840
20	M+H-C18:1	0.11 ± 0.00	/	861.6397	[M+H]^+^	579.3840
21	M+H-C18:0	4.91 ± 0.24	/	863.6554	[M+H]^+^	579.3840
22	M+H-C20:2	2.27 ± 0.12	/	883.6241	[M+H]^+^	579.3840
23	M+H-C20:1	1.41 ± 0.15	/	885.6397	[M+H]^+^	579.3840
24	M+H-C20:0	0.56 ± 0.03	/	887.6554	[M+H]^+^	579.3840
25	M+H-C18:4/C18:4	0.75 ± 0.03	/	1113.7906	[M+H]^+^	860.6783
26	M+H-C18:4/C18:3	1.39 ± 0.05	/	1115.8062	[M+H]^+^	862.6959
27	M+H-C18:3/C18:3	0.90 ± 0.12	/	1117.8219	[M+H]^+^	870.7575
28	M+H-C18:2/C18:3	0.19 ± 0.00	/	1119.8375	[M+H]^+^	872.7743
39	M+H-C18:1/C18:3	2.24 ± 0.09	/	1121.8532	[M+H]^+^	874.7896
30	M+H-C18:1/C18:1	0.44 ± 0.10	/	1125.8845	[M+H]^+^	888.7703

*m/z*: ratio of an ion’s mass (m) in atomic mass units (z) to its formal charge (z); M: molecule with molecular weight m; Adduct: product of a direct; LOQ: the values of the identified compounds did not reach the limit of quantification.

## Data Availability

Data available upon request from jana.ambrozic@um.si.
